# A new generation of trade policy: potential risks to diet-related health from the trans pacific partnership agreement

**DOI:** 10.1186/1744-8603-9-46

**Published:** 2013-10-16

**Authors:** Sharon Friel, Deborah Gleeson, Anne-Marie Thow, Ronald Labonte, David Stuckler, Adrian Kay, Wendy Snowdon

**Affiliations:** 1 National Centre for Epidemiology and Population Health, The Australian National University, Acton 0200, ACT, Australia; 2 School of Public Health and Human Biosciences, La Trobe University, Melbourne, VIC 3086, Australia; 3 Menzies Centre for Health Policy, School of Public Health, University of Sydney, Darlington, NSW 2006, Australia; 4 Institute of Population Health, University of Ottawa, Ottawa ON K1N 6N5, Canada; 5 Department of Sociology, University of Cambridge, Cambridge, UK; 6 Crawford School of Public Policy, The Australian National University, Acton, ACT 0200, Australia; 7 C-POND, Fiji National University and Deakin University, Suva, Fiji

**Keywords:** Trade policy, Free trade agreements, Investment treaties, Food and nutrition, Health inequity

## Abstract

Trade poses risks and opportunities to public health nutrition. This paper discusses the potential food-related public health risks of a radical new kind of trade agreement: the Trans Pacific Partnership agreement (TPP). Under negotiation since 2010, the TPP involves Australia, Brunei, Canada, Chile, Japan, Malaysia, Mexico, New Zealand, Peru, Singapore, the USA, and Vietnam. Here, we review the international evidence on the relationships between trade agreements and diet-related health and, where available, documents and leaked text from the TPP negotiations. Similar to other recent bilateral or regional trade agreements, we find that the TPP would propose tariffs reductions, foreign investment liberalisation and intellectual property protection that extend beyond provisions in the multilateral World Trade Organization agreements. The TPP is also likely to include strong investor protections, introducing major changes to domestic regulatory regimes to enable greater industry involvement in policy making and new avenues for appeal. Transnational food corporations would be able to sue governments if they try to introduce health policies that food companies claim violate their privileges in the TPP; even the potential threat of litigation could greatly curb governments’ ability to protect public health. Hence, we find that the TPP, emblematic of a new generation of 21st century trade policy, could potentially yield greater risks to health than prior trade agreements. Because the text of the TPP is secret until the countries involved commit to the agreement, it is essential for public health concerns to be articulated during the negotiation process. Unless the potential health consequences of each part of the text are fully examined and taken into account, and binding language is incorporated in the TPP to safeguard regulatory policy space for health, the TPP could be detrimental to public health nutrition. Health advocates and health-related policymakers must be proactive in their engagement with the trade negotiations.

## 21st century trade: radical change and real concern for public health

Trade agreements pose a major risk for food insecurity and nutrition-related disease [1-3]. The suite of multilateral trade agreements initiated by the World Trade Organization (WTO) and subsequently deepened through an increasing number of bilateral and regional trade agreements (RTAs), have brought about three important changes to food systems: opening of domestic markets towards international food trade and foreign direct investment (liberalisation); subsequent increased entry of transnational food companies and their global market (integration), and global food advertising (cultural hybridization). These three changes affect population diets, and raise concerns about undernutrition, obesity and non-communicable diseases, by altering the local availability, nutritional quality, price and desirability of foods [2,4,5].

This paper aims to alert policy-makers, researchers and non-government organisations to the potential harmful impacts of new forms of free trade agreements, exemplified by the Trans Pacific Partnership (TPP) agreement, on nutrition and diet-related health. The TPP has been under negotiation since 2010, and involves Australia, Brunei, Canada, Chile, Japan, Malaysia, Mexico, New Zealand, Peru, Singapore, the USA and Vietnam [6], with more likely to accede over time.

A radical new generation of RTAs are emerging in the 21st century, extending the scope and reach of trade agreements. In theory, trade liberalisation - the reduction in barriers to trade - can improve economic growth through increased export opportunities to overseas markets, attraction of foreign investment into countries and lower cost imported goods [7]. Trade-generated income can potentially benefit population health and nutrition by improving access to healthcare, labour standards and quality and quantity of food [8-10]. However, trade liberalisation is known to create winners and losers between and within countries [11-15]; with the ‘trickle down’ social and health benefits depending partly on the progressivity of a country’s regulatory and redistributive policies [16]. Trade is no longer simply about the exchange of raw materials and final goods between countries. It has evolved into a complex “trade-investment-service nexus”, involving integrated flows of goods, services, people, ideas, and investments in physical, human and knowledge capital [17]. These developments affect policymakers’ control over regulating their economies and have implications for how the theorised benefits of liberalised trade are distributed, but importantly can also affect the policy space governments have for health or social purposes [18]. Multilateral trade is governed by rules set through the World Trade Organisation (WTO) [19]. As the returns for high income countries from multilateral trade decreased over time, with stalemates between countries in the multilateral Doha development agenda, and the economic power balance shifted between countries and regions, increasing numbers of regional trade agreements (RTAs) and bilateral investment treaties emerged, sitting outside the multilateral trading rules [17,20]. All members of the WTO, when negotiating a trade agreement, must adhere to its rules on bilateral and regional trade agreements. Although the WTO’s “enabling clause” recognises that developing countries may need to protect some, or many, sectors of their economies from open competition with other countries’ exporters, the clause *does not* extend to regional trade agreements involving developed countries. The number and scope of RTAs are increasing rapidly [21]. The WTO’s analysis of the types of provisions of 97 RTAs globally found that four appear in over a third of RTAs, but which are not part of the WTO’s rulebook. These are competition policy, movement of capital, IPRs not in the multilateral TRIPs Agreement, and investment liberalisation. Known as the 1996 Singapore Issues, developing countries ruled these four issues off the agenda for the multilateral Doha Development Round. Since no agreement was reached to include these issues through the WTO, developed nations have pushed that any new bilateral or regional trade negotiations in which they enter must include these issues. None of these provisions are tariff-based, but instead are regulatory in nature, and their growth is “testimony to the growing importance of behind-the-border measures in RTAs” [17].

The TPP is an RTA unlike any of its predecessors [22]. It is a misnomer to call it a trade agreement: The TPP will be more like an investment treaty, designed to increase economic integration and arguably shifting the balance of policy-making power firmly in favour of corporate interests [23]. Whilst it would include traditional measures, based on what we know from leaked documents and public stakeholder consultations, the TPP appears to have unprecedented protections for investors and intellectual property rights holders [8]. Upon signing the agreement, changes to domestic policies are also likely to be required in relation to regulatory coherence, transparency, trade facilitation and harmonisation. These ‘behind-the-border’ regulatory controls on government increasingly limits policy space and national sovereignty to regulate investors or introduce public health policies that investors consider in contravention to the trade agreement [24-26].

TPP negotiations are taking place under conditions of confidentiality. While the broad outlines of the TPP were announced at the Asia Pacific Economic Cooperation meeting in November 2011, analysis must rely on leaked documents including draft text leaked from the negotiations [publically available from a range of websites including http://www.citizen.org; http://tinyurl.com/tppinvestment; http://www.infojustice.org/archives/category/trade-agreements/trans-pacificpartnership; http://keionline.org/tpp], updates from trade departments [http://www.dfat.gov.au/fta/tpp/index.html], and the US Congressional Research Service reports available after every negotiation round [http://fpc.state.gov/c18185.htm], and discussions with trade negotiators who have spoken publically through the stakeholder consultations about the broad form of the TPP. However, given the lack of available official information, and the ongoing nature of negotiations, this paper is necessarily exploratory in nature. First we review the evidence of the impact of existing trade agreements, particularly RTAs, on population nutrition globally. Informed by this, plus analysis of leaked information relating to the TPP, we hypothesise ways in which the TPP may pose risks to nutrition.

## Pathways from trade to diet-related health: lessons from previous trade agreements

To investigate the relationships between existing trade agreements, nutrition and diet-related health, a literature search of a cross-disciplinary range of databases was undertaken, including: Science Direct, PubMed, ProQuest,SpringerLink, and Google Scholar. The grey literature was searched and reports from government and non-government organisations were used.

**Figure 1:**
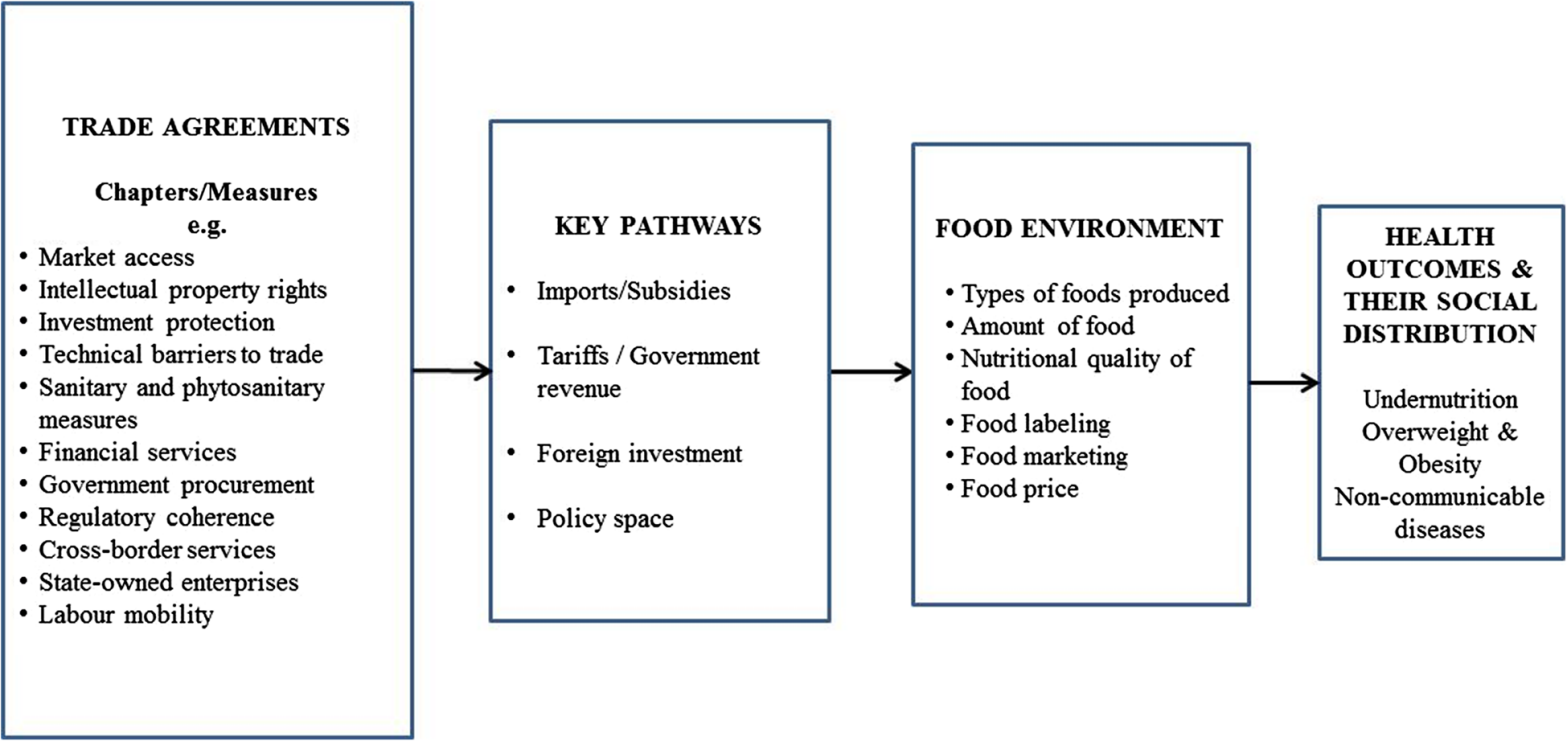
Conceptual framework of the relationships between trade agreements, food environments and diet-related health.

A conceptual overview of the relationship between trade, food and diet-related health is shown in Figure 1. The component parts of trade agreements are called ‘chapters’. In developing a trade agreement, the provisions are negotiated for each chapter. There are three major pathways through which trade agreements can affect nutrition, each of which is now discussed.

### Imports: access to nutritious foods

Trade liberalisation has traditionally focused on reductions in tariff and non-tariff barriers to trade. This can lead to greater amounts and types of food being imported into countries, which in turn can alter the nutritional quality, amount and price of food available, thus shaping food preferences and affecting diet-related health [2,27,28]. While reductions in barriers to trade can increase consumer food choices, and improve supply for net-food importing countries, trade liberalisation has resulted in disproportionately large increases in imports and domestic production of processed foods, skewing the food supply towards an over-supply of highly processed foods that are calorie-rich and nutrient-poor [2,29-31].

In Central America, increased imports promoted greater availability of foods associated with the nutrition transition, such as meat, dairy products and processed foods [31]. The ratification of the Central America-USA Free Trade Agreement (CAFTA) in 2006 furthered this liberalisation through agreements on tariffs and sanitary and phytosanitary regulations [32], and is predicted to expand trade in meat and processed food. Similar trends have been observed with the lowering of trade barriers between Mexico and the USA following the signing of the North American Free Trade Agreement (NAFTA):imports of corn, soybeans, sugar, snack foods, and meat products into Mexico increased significantly [33]. Trade liberalisation has driven the nutrition transition in the Pacific Island countries (PICs), particularly by increasing fat consumption through imports of vegetable oils, margarine, butter, meat, chickens and canned meat [34-38]. Between 1963 and 2000, the total fat supply in PICs increased by as much as 80% [38].

### Tax revenues and government spending

Tariff-reduction can affect nutrition through its potential to reduce the tax-raising capability of governments to fund health programs [39]. For example, a reduction in tariffs under the proposed PACER Plus agreement, which includes Australia, New Zealand and the PICs, could affect tax revenues for social spending in the PICs. Participating countries would need to adhere to the WTO’s GATT Article XXIV requiring the elimination of “substantially all” import tariffs. Projections suggest a revenue loss for many PICs’ governments, equivalent to a significant proportion of their education or health budgets [39].

### Increasing foreign direct investment and integrated food supply chains

An increasingly important aspect of trade policy is investment liberalisation, intended to facilitate foreign direct investment (FDI) by international companies. The link between trade and NCDs comes partly through the global diffusion of food products that are harmful to health [40,41] enabled by greater investment and penetration of transnational food corporations (TNCs) into many developing countries. FDI is a key strategy used by TNCs to extend their supply chains (production, processing, distribution and marketing, placing control over all parts of the global supply chain into the hands of a relatively small number of TNCs [42]. In Mexico, NAFTA has enabled significant US agribusiness investment across the full spectrum of the food supply chain [33], creating challenges for local agriculture production, changing the focus of production from domestic to export cash crop production.

There has also been an emergence of global food processors and retailers such as Unilever, Nestle, Wal-Mart, Carrefour, and Tesco. These TNCs, especially the supermarkets, influence eating habits through the products they choose to sell, the retail price, and the labelling and promotion of particular goods [43]. Increasing market penetration by TNCs has led to a dramatic increase in the transfer of highly processed foods from developed to developing countries, creating national marketplaces crammed with cheap nutrient-poor foods [2,44].

In Central America, reductions in barriers to investment were critical in the expansion of highly processed food markets [31]. These trends have been supported by further liberalisation under CAFTA, with an expected increased production of processed foods by US companies based in Central America, as well as by domestic companies (due to a more competitive market environment) [32]. Similarly, investment in Fiji by TNCs has increased availability and consumption of processed foods [37]. Stuckler *etal* have shown that FDI liberalisation through trade agreements with the USA significantly increased the consumption of soft drinks within the signatory country, consequently increasing the risk of some NCDs [45].

In 2006, when Thailand proposed on public health grounds the introduction of a front of pack traffic light labelling system on snack food products, many of which had been introduced into the country by US-owned TNCs [44], the USA and other countries claimed that contravened the Agreement on Technical Barriers to Trade [46]. The Thai government abandoned the traffic light system and implemented a monochrome Guideline Daily Amounts label [47], a decision widely regarded as reflecting the interests of the food industry.

## Why the TPP poses new concerns for nutrition and diet-related health

Based on the above analysis of existing trade agreements, and following assessment of government documents and draft text describing the likely provisions within the different TPP chapters, we now hypothesise ways in which the TPP could affect nutrition and health.

At the time of writing, there are twenty-nine TPP chapters under negotiation. The likely chapters with most relevance to nutrition-related health include: Market Access; Intellectual Property Rights (IPRs); Cross-border Services; Technical Barriers to Trade (TBT); Sanitary and Phytosanitary measures (SPS); Investment; and Dispute Resolution and Transparency (all of which have precedents in WTO agreements); and new chapters on Competition and State-owned Enterprises (SOEs); Regulatory Coherence; and Government Procurement (presently an optional agreement within the WTO).

In addition to the nutrition issues associated with existing FTAs, as raised in the previous section, the new health concerns with the TPP arise from the intensification of existing trade provisions and unprecedented protections for investors and intellectual property rights-holders [8]. Under WTO trade rules, any FTA must offer at a minimum the same WTO provisions. The only reason to include chapters on topics that already exist in WTO agreements into new trade agreements is to intensify them, requiring deeper liberalisation commitments or more extensive IP protection. A more novel concern is that the TPP appears to include various unprecedented provisions which would increase the privileges of TNCs and potentially reach much further into the regulation of domestic public policy that any previous FTA [48].

### More and easier foreign investment: facilitating supply chain integration

As Kelsey noted at the Global Alcohol Policy conference [49], grocery manufacturers are pressing for greater integration and streamlining of policies and regulations around the movement of food and beverages on the basis that their supply chains are increasingly global and that without this streamlining trade is expensive and complicated [49]. A central aim of the TPP is to support more FDI by food companies and consolidation of ownership along the food supply chain in the region.

Some example TPP chapters include the *Investment* chapter. The multilateral Agreement on Trade Related Investment Measures (TRIMs) contains rules that apply to the domestic regulations a country applies to foreign investors. The TPP *Investment* chapter may extend the provisions in TRIMs, creating a business climate even more conducive to long-term investment by the transnational food industry.

The food industry’s strategy of concentrating on marketing highly processed premium products on a global scale requires minimal regulatory variation on advertising and labelling, with unimpeded access across the entire range of media. If the rules in the *Cross*-*border Services* chapter in the TPP prevent national governments from limiting access to and the growth of their markets in a particular sector, or if they prohibit limits on quantity or size of services operations, this could enable greater access and increasing growth of their markets by food companies producing and selling highly processed foods that are associated with obesity and NCDs.

The Agreement on Government Procurement is optional in the WTO, and few developing countries have signed up to it. Its inclusion appears to be a mandatory provision in the TPP *Government Procurement* chapter, and essentially means that, depending on the exceptions or limitations placed on this chapter, government tenders will have to be open to bids from companies in any country that signs on to the TPP, and the conditions governments place on their tendering processes could be reduced. Depending on what is covered by the Government Procurement chapter of the TPP, governments contracting for food services (e.g. in their schools, their hospitals, their cafeterias) may have less control over the nutritional quality (and certainly the geographic origins) of the food being offered.

### Encroachment on policy space: protecting the investor at the cost of public health

In addition to influencing the nutritional quality and price of foods available in countries, many TPP chapters would likely reduce the regulatory flexibilities that governments retain within the multilateral WTO agreements. By doing so, this could undermine health policy goals and extend the control of the food industry over domestic policy-making.

Food industry influence on public policy development is already a significant problem in many countries [3]. A number of the proposed TPP chapters appear to contain provisions that would increase the role of the food industry in policy making. One example is the *Regulatory Coherence* chapter. If this requires, as is suggested, the establishment of a central mechanism or body to coordinate the development of policy, it could provide a venue for industry input into regulatory decision making, which is not a good thing for health and nutrition goals [23].

Some of the policy space available with respect to IPRs under the TRIPS multilateral trade rules could be challenged in the TPP negotiations. If the *Intellectual Property* chapter constrains governments’ ability to regulate food advertising and labelling, communities including children could be exposed to the marketing of highly processed food products.

If the *Technical Barriers to Trade* chapter intensifies the provisions in the multilateral TBT agreement, and seeks to ensure fewer “trade restrictive” measures related to food products, this may impact governments’ ability to regulate food labelling of highly processed foods.

The proposed *Investment* chapter of the TPP is highly problematic for public health as it would give investors the right to sue governments and demand compensation for post-TPP changes in domestic financial, health, environmental, and other laws that investors claim undermine their new TPP privileges or the value (‘expropriation’) of their investments generally. ‘Investments’ have been defined broadly in other RTAs, and in the TPP are likely to include trademarks, shares in or ownership of an entity, licenses to manufacture or sell food products, and distribution agreements. The TPP definition of ‘expropriation’ will likely be at least as broad as that adopted in other RTAs such as NAFTA, which was considerably more broad than the national legal definitions found in two of its three country members. Also, the TPP Investment chapter may not include the WTO’s General Agreement on Trade in Services exceptions for measures “necessary to protect human, animal or plant life and health” [50].

The *Investor*-*State Dispute Settlement* (ISDS) mechanism proposed by the USA would enable foreign corporations to sue governments if they try to regulate the food industry in such a way as to reduce the value of their investment (e.g. by introducing labelling requirements and advertising restrictions). The investor-state arbitration process lacks many of the safeguards of domestic legal processes and has several fundamental flaws. Cases are decided by a panel of three arbitrators, who may also represent corporations in concurrent cases, creating an inherent pro-investor bias [51]. Hearings frequently lack transparency, and arbitration costs can amount to hundreds of millions. Even the possibility of arbitration may be a significant deterrent to governments.

### Viewing the TPP holistically

The protection of investors’ rights and changes to domestic policy instruments occurs in multiple TPP chapters. For example, provisions in the regulatory coherence and transparency chapters appear to tightly specify how policy should be made, and may interact with the investment chapter to provide further grounds for investor-state disputes [52]. It is important therefore to view the TPP holistically and the links between various chapters in the TPP considered together to fully understand the potential impact on nutrition and health.

## Conclusions

Prioritisation by the TPP of investors and the associated controls on policy-making would raise legitimate concerns for population nutrition. The TPP could include changes to domestic regulatory regimes facilitating greater industry input to policy processes and more avenues of appeal, more policy controls with implications for regulation of foreign investment in domestic food production and retailing, and extensive IPRs which could affect food labelling and advertising restrictions. The net effect of these changes would be to strengthen the influence of mainly western TNCs on government policy, and weaken public health’s ability to protect populations against unhealthy commodities. These risks and impacts will not be experienced equally between countries or social groups, thereby exacerbating nutrition and health inequities.

Due to the secrecy of the negotiations it is unclear if there are safeguards in place for diet-related health but there does not appear to be any systematic consideration of health concerns in the negotiations. Rebalancing the influence of food corporations in the TPP negotiation processes with input from the health sector is vital. Public health nutrition advocates and health-related policymakers must be proactive in their engagement with the trade negotiations to minimise negative outcomes and preserve policy space for population nutrition goals. A window of opportunity exists to integrate these concerns into the TPP while it remains under negotiation. Public health advocates could be contributing to stakeholder forums, meeting with trade officials, and lobbying Ministers for Health to engage with the trade negotiations. These short term interventions are not enough. Evidence to inform trade policy that embeds principles of health, nutrition and equity, and implementation strategies that mitigate the negative health consequences of the TPP and other trade agreements are needed.

## Abbreviations

TPP: The trans pacific partnership agreement; WTO: The world trade organization; RTAs: Regional trade agreements; CAFTA: Central America-USA free trade agreement; NAFTA: North American free trade agreement; PICs: Pacific island countries; FDI: Foreign direct investment; TNCs: Transnational food corporations; IPRs: Intellectual property rights; TBT: Technical barriers to trade; SPS: Sanitary and phytosanitary measures; SOEs: Competition and state-owned enterprises; GATS: General agreement on trade in services; TRIMs: Trade related investment measures; ISDS: Investor-state dispute settlement.

## Competing interests

Sharon Friel is a member of the editorial board of the journal Globalization and Health.

## Authors’ contributions

SF conceived the piece and wrote the initial draft. All authors contributed to the conceptual development and writing of all parts of the manuscript. All authors read and approved the final manuscript.

## References

[B1] Hawkes C Murphy S Hawkes C, Blouin C, Henson S, Drager N, Dube L An overview of global food trade Trade, Food, Diet and Health Perspectives and Policy Option 2010 Oxford: John Wiley & Sons Inc

[B2] Hawkes C Chopra M Friel S Labonte R, Schrecker T, Packer C, Runnels V Globalization, trade and the nutrition transition Globalization and Health: Pathways, Evidence and Policy 2009 New York: Routledge

[B3] Stuckler D Nestle M Big food, food systems, and global health PLos Med 2012 9 e1001242 10.1371/journal.pmed.1001242 22723746 PMC3378592

[B4] FAO The state of food insecurity in the world, 2008 : high food prices and food security: threats and opportunities 2008 Rome: Food and Agriculture Organization of the United Nations (FAO)

[B5] Friel S Baker PI Equity, food security and health equity in the Asia Pacific region Asia Pac J Clin Nutr 2009 9 620 632 19965356

[B6] Office of the United States Trade Representative Enhancing Trade and Investment, Supporting Jobs, Economic Growth and Development: Outlines of the Trans-Pacific Partnership Agreement 2011 Washington DC: Office of the United States Trade Representative

[B7] Makki SS Somwaru A Impact of foreign direct investment and trade on economic growth: evidence from developing countries Am J Agric Econ 2004 9 795 801 10.1111/j.0002-9092.2004.00627.x

[B8] World B World Development Report 2010: Development and Climate Change 2010 Washington DC: World Bank

[B9] World Bank World Development Report 2006: Equity and Development 2005 Washington: World Bank

[B10] Bank W World development report 2008: Agriculture for Development 2008 Washington, DC: World Bank

[B11] Stiglitz J Making Globalization Work 2006 New York: WW Norton and Company

[B12] Stiglitz JE Trade agreements and health in developing countries Lancet 2009 9 363 365 10.1016/S0140-6736(08)61772-9 19167055

[B13] Labonte R Schrecker T Packer C Runnels V Globalization and Health: Pathways, Evidence and Policy 2009 New York: Routledge

[B14] CSDH Closing the gap in a generation: health equity through action on the social determinants of health. Final report of the Commission on Social Determinants of Health 2008 Geneva: World Health Organisation 10.1016/S0140-6736(08)61690-6 18994664

[B15] Smith RD Lee K Drager N Trade and health: an agenda for action Lancet 2009 9 768 773 10.1016/S0140-6736(08)61780-8 19167056 PMC2726935

[B16] Labonté R Blouin C Forman L Kay A, Williams O Trade and health Global Health Governance: Crisis, Institutions and Political Economy 2009 London: Palgrave Macmillan

[B17] Baldwin R21st century regionalism: Filling the gap between 21st century and 20th century trade rules. Policy Insights 2011 No.56 2011 Geneva: Centre for Economic Policy Research

[B18] Khor M Bilateral/Regional Free Trade Agreements: An outline of elements, nature and development implications 2005 Penang: Third World Network

[B19] Labonte R Schrecker T Introduction: Globalization’s Challenges to People’s Health Globalization and health: pathways, evidence and policy 2009 New York: Routledge

[B20] Baldwin R Thornton P Multilateralising Regionalism: Ideas for a WTO Action Plan on Regionalism 2009 London: Centre for Economic Policy Research

[B21] UNESCAP Asia-Pacific Trade and Investment Report 2011: Post-crisis trade and investment opportunities 2011 Bangkok: United Nations Economic and Social Commission for Asia and the Pacific

[B22] Schott J Kotschwar B Muir J Understanding the Trans-Pacific Partnership 2013 Washington DC: Peterson Institute for International Economics

[B23] Kelsey J New-generation free trade agreements threaten progressive tobacco and alcohol policies Addiction 2012 9 1719 1721 10.1111/j.1360-0443.2012.03874.x 22404183

[B24] Gleeson D Legge D Strengthening public health engagement in trade policy: PHAA’s policy on trade agreements and health Aust N Z J Public Health 2012 9 7 9 10.1111/j.1753-6405.2012.00823.x 22313698

[B25] Gleeson D Friel S Emerging threats to public health from regional trade agreements Lancet 2013 9 9876 1507 1509 23453142 10.1016/S0140-6736(13)60312-8

[B26] Faunce T Townsend R The Trans Pacific Partnership Agreement: challenges for Australian health and medicine policies Med J Aust 2011 9 83 86 21241222 10.5694/j.1326-5377.2011.tb04174.x

[B27] Lock K Stuckler S Charlesworth K McKee M Rising global food prices: potential causes and health impacts Br Med J 2009 9 269 272 10.1136/bmj.b2403 19596718

[B28] Kay A Williams O The international political economy of global health governance 2009 Basingstoke: Palgrave Macmillan

[B29] Blouin C Chopra M van der Hoeven R Trade and social determinants of health Lancet 2009 9 502 507 10.1016/S0140-6736(08)61777-8 19167058

[B30] Thow AM Trade liberalisation and the nutrition transition: mapping the pathways for public health nutritionists Public Health Nutr 2009 9 2150 2158 10.1017/S1368980009005680 19433005

[B31] Thow AM Hawkes C The implications of trade liberalization for diet and health: a case study from Central America Glob Heal 2009 9 5 10.1186/1744-8603-5-5 PMC2729306 19638196

[B32] Hawkes C Thow AM Implications of the Central America-Dominican Republic-free trade agreement for the nutrition transition in Central America Rev Panam Salud Publica 2008 9 345 360 10.1590/S1020-49892008001100007 19141177

[B33] Clark SE Hawkes C Murphy SME Hansen-Kuhn KA Wallinga D Exporting obesity: US farm and trade policy and the transformation of the Mexican consumer food environment Int J Occup Environ Health 2012 9 53 65 10.1179/1077352512Z.0000000007 22550697

[B34] Thow A Snowdon W Hawkes C, Blouin C, Henson S, Drager N, Dubé L The effect of trade and trade policy on diet and health in the Pacific Islands Trade, Food, Diet and Health: Perspectives and Policy Options 2010 Oxford: Wiley Blackwell

[B35] Legge D Gleeson D Snowdon W Trade Agreements and Non-communicable Diseases in the Pacific Islands 2011 Fiji: Pacific NCD Forum

[B36] Snowdon W Moodie M Schultz J Swinburn B Modelling of potential food policy interventions in Fiji and Tonga and their impacts on noncommunicable disease mortality Food Policy 2011 9 597 605 10.1016/j.foodpol.2011.06.001

[B37] Thow A-M Heywood P Schultz J Quested C Jan S Colagiurie S Trade and the nutrition transition: strengthening policy for health in the pacific Ecol Food Nutr 2011 9 18 42 10.1080/03670244.2010.524104 21888586

[B38] Hughes R Lawrence M Globalization, food and health in Pacific Island countries Asia PacJClinNutr 2005 9 298 306 16326635

[B39] Oxfam PACER Plus and its Alternatives: Which way for trade and development in the Pacific? Oxfam briefing paper 2009 2009 Auckland: Oxfam Australia and Oxfam New Zealand

[B40] Labonte R Mohindra K Lencucha R Framing international trade and chronic disease Glob Heal 2011 9 doi:10.1186/1744-8603-1187-1121 10.1186/1744-8603-7-21 PMC3158109 21726434

[B41] Friel S Chopra M Satcher D Unequal weight: equity oriented policy responses to the global obesity epidemic BMJ 2007 9 1241 1243 10.1136/bmj.39377.622882.47 18079548 PMC2137064

[B42] Lang T Heasman M Food wars : the global battle for minds, mouths, and markets 2004 Earthscan: London, Sterling, VA

[B43] Dixon J Germov J, Williams L Adding Value(s): A cultural economy analysis of supermarket power A sociology of food and nutrition: the social appetite 2004 Melbourne: Oxford University Press 96 116

[B44] Hawkes C The role of foreign direct investment in the nutrition transition Public Health Nutr 2005 9 357 365 15975180 10.1079/phn2004706

[B45] Stuckler D McKee M Basu S Global saturation of “risky commodities”: the role of global producers in increased population consumption of processed food, tobacco, and alcohol PLoS Med 2012 9 e1001235 10.1371/journal.pmed.1001235 22745605 PMC3383750

[B46] WTO Minutes of the meeting of 21 March 2007, Committee on Technical Barriers to Trade 2007 Geneva: World Trade Organization

[B47] Sirikeratikul S Vasquez O Thai FDA’s New Guideline Daily Amounts (GDA) Labeling 2011 Washington DC: United States Department of Agriculture Foreign Agricultural Service

[B48] Kelsey J Investment developments in the Trans-Pacific Partnership agreement Investment Treaty News 2012 Winnipeg: International Institute for Sustainable Development

[B49] Kelsey J The implications of new generation free trade agreements for alcohol policies Conference paper: Global Alcohol Policy conference 2012 Nonthaburi, Thailand:

[B50] Public Citizen Public Interest Analysis of Leaked Trans-Pacific Partnership (TPP) Investment Text 2012 http://www.citizen.org/trade/

[B51] Gleeson D Tienhaara K Faunce T Challenges to Australia’s national health policy from trade and investment agreements Med J Aust 2012 9 1 3 10.5694/mja11.11635 22432677

[B52] Kelsey J Preliminary analysis of the draft TPP chapter on domestic coherence 2011 http://www.citizenstrade.org/ctc/wp-content/uploads/2011/10/TransPacific_RegCoherenceMemo.pdf

